# Methods and Software Tools for Reliable Operation of Flying LiFi Networks in Destruction Conditions

**DOI:** 10.3390/s24175707

**Published:** 2024-09-02

**Authors:** Herman Fesenko, Oleg Illiashenko, Vyacheslav Kharchenko, Kyrylo Leichenko, Anatoliy Sachenko, Lukasz Scislo

**Affiliations:** 1 Department of Computer Systems, Networks and Cybersecurity, National Aerospace University “KhAI”, 17, Chkalov Str., 61070 Kharkiv, Ukraine; h.fesenko@csn.khai.edu (H.F.); v.kharchenko@csn.khai.edu (V.K.); k.leychenko@csn.khai.edu (K.L.); 2 The Institute of Informatics and Telematics of the National Research Council (IIT-CNR), Research Area Via Giuseppe Moruzzi, 1, 56124 Pisa, Italy; 3 Research Institute for Intelligent Computer Systems, West Ukrainian National University, 11, Lvivska Str., 46009 Ternopil, Ukraine; as@wunu.edu.ua; 4 Department of Informatics and Teleinformatics, Kazimierz Pulaski University of Radom, ul. Malczewskiego 29, 26-600 Radom, Poland; 5 Faculty of Electrical and Computer Engineering, Cracow University of Technology, 24, Warszawska, 31-155 Cracow, Poland

**Keywords:** unmanned aerial vehicle, flying LiFi network, production facility, sensors, obstacles, planning stages, stationary depot, reliability, software tools

## Abstract

The analysis of utilising unmanned aerial vehicles (UAVs) to form flying networks in obstacle conditions and various algorithms for obstacle avoidance is conducted. A planning scheme for deploying a flying LiFi network based on UAVs in a production facility with obstacles is developed and described. Such networks are necessary to ensure reliable data transmission from sensors or other sources of information located in dangerous or hard-to-reach places to the crisis centre. Based on the planning scheme, the following stages are described: (1) laying the LiFi signal propagation route in conditions of interference, (2) placement of the UAV at the specified points of the laid route for the deployment of the LiFi network, and (3) ensuring the reliability of the deployed LiFi network. Strategies for deploying UAVs from a stationary depot to form a flying LiFi network in a room with obstacles are considered, namely the strategy of the first point for the route, the strategy of radial movement, and the strategy of the middle point for the route. Methods for ensuring the uninterrupted functioning of the flying LiFi network with the required level of reliability within a given time are developed and discussed. To implement the planning stages for deploying the UAV flying LiFi network in a production facility with obstacles, the “Simulation Way” and “Reliability Level” software tools are developed and described. Examples of utilising the proposed software tools are given.

## 1. Introduction and Related Works

### 1.1. Motivation

In the event of accidents at critical infrastructure facilities, the crisis centre (CC) personnel need to receive information about the main parameters of the technological equipment and the level of working environment contamination inside the production premises. However, due to the accident, the regular communication equipment may be damaged, and the above-mentioned important information will cease to reach the CC, thus making it much more difficult for its personnel to make effective management decisions regarding the response to the accident. In this case, a flying LiFi network based on UAVs can be deployed to renew the flow of information from sensors or other data sources located in dangerous or hard-to-reach places to the CC.

The use of UAVs to form a LiFi network is because they provide flexibility to the LiFi network in deployment scenarios, especially those that involve deployment in various environments where traditional radio frequency communication may be problematic, such as manufacturing facilities with destruction. Such versatility allows this network to perform multiple tasks in various operating conditions.

The flying LiFi network is immune to electromagnetic interference from other electronic devices or radio frequency (RF) signals. It can offer significantly higher rates of secure data transfer than traditional RF communication technologies. This property becomes especially important for scenarios involving the fast transfer of large amounts of data (streaming high-definition video, real-time data transmission from sensors) or large-scale video surveillance.

However, it should be noted that light signals can be limited by obstacles in destroyed/damaged production premises. This will require laying rational routes for the propagation of the LiFi signal to bypass these obstacles, determining the locations of UAVs on these routes, and ensuring the guaranteed functioning of the flying LiFi network during the given time.

Thus, developing methods and software tools to support deploying unmanned flying LiFi networks to ensure data transmission in destruction conditions is a hot scientific and applied issue, given the requirements for time and reliability characteristics.

### 1.2. State of the Art

*UAV systems in confined spaces and complex environments*. More and more research has been conducted using UAVs for data transmission in confined spaces and complex cyber-physical environments. For example, the authors of [1] propose approaches for deploying a UAV system in difficult indoor conditions without possibly using a Global Positioning System (GPS) for internal inspections of pipelines and boilers. A system consisting of the hardware description and integration of two UAVs, a two-stage simultaneous localisation and mapping method for UAV localisation and 3D environmental mapping, a safety-guaranteed trajectory planning algorithm for surveying and data collection, and an obstacle-aware trajectory generation method are proposed. The study of [2] presents an approach to localising a UAV indoors using a quasi-tensioned cable. It notes that the approach can reduce the error of modern cable-based indoor localisation by 31.12%. Since the UAV is localised relative to the centre of the cable reel, the method can be used to localise the UAV in a moving frame. Therefore, it is suitable for mutual localisation in marsupial heterogeneous robotic teams for search and rescue operations in urban areas. The work of [3] considers analytical models of a cooperative satellite–UAV communication system, where UAVs act as aerial repeaters using free-space optical technologies. The authors of [4] devote research to the problem of physical layer functioning in satellite–UAV communication systems from three points of view: time-varying channel estimation, high-mobility transceiver, and high-mobility multiple access. Relevant key technologies, existing works, open problems, and further development directions are presented and considered.

*UAV motion and path planning*. The work of [5] describes the development and comparison of two UAV motion planning algorithms with the possibility and impossibility of contact with the environment. The authors propose two motion planning schemes that reduce the bound-aware reachable configuration space, deliberately plan (and weaken) the points of contact of the bound with the environment, and provide an equivalent reachable configuration space as the unbound counterpart. The authors emphasise that their approach allows tethered aircraft to be used in confined and cluttered environments with obstacles instead of ideal free space while retaining the benefits of tethering. The authors of [6] offer a set of algorithms for planning the UAV flight path indoors, optimised for difficult conditions. Firstly, an algorithm for optimised indoor air space grid modelling based on a grid of geographic coordinates division with one-dimensional integral coding on a 2ⁿ-tree in three dimensions is proposed to reduce the computational complexity of modelling three-dimensional air space in the room using spatial subdivisions. The authors of [7] provide an overview of proposals for the architecture of obstacle avoidance and UAVs. Different approaches are usually considered, including sensor-based, planning, and control approaches. Various network architectures, such as peer-to-peer, centralised, and hybrid networks, are also discussed. The authors conclude that obstacle avoidance algorithms and perfect network architecture are the two most important aspects of developing reliable and efficient UAVs. They point out that there are many different approaches to obstacle avoidance and network architecture, and the choice of the appropriate approach depends on the specific application of the UAV. Recently, more and more attention has been paid to solving problems related to obstacle avoidance and route planning for ground and air robots. For example, the authors of [8] apply the Delaunay triangulation algorithm and the improved A* algorithm to analyse complex obstacles and generate Voronoi points as priority nodes for path finding to improve the efficiency of mobile robot trajectory planning. First of all, the authors build a triangular grid based on a set of obstacles and nodal points. The corner points are then transformed into a graph used in the improved A* algorithm to find the shortest path between the start and endpoints. First of all, it should be noted that the algorithm is resistant to changes and simple to implement. However, the algorithm may be less efficient in austere environments, lose efficiency in dynamic environments where changes occur rapidly, and be computationally expensive for large environments. In the works of [9,10], a new approach to dynamic path planning for mobile robots is presented based on a hybrid solution of an ant colony and dynamic windows. The ant colony algorithm is a biologically inspired search algorithm that mimics the foraging behaviour of ants. The dynamic window algorithm is a real-time path planning algorithm that considers the robot’s current state and dynamic changes in the environment. The proposed approach combines the advantages of the ant colony algorithm and the dynamic window algorithm to create a more efficient and reliable obstacle avoidance route planning algorithm. The ant colony algorithm finds a global path to the goal, and the dynamic window algorithm generates a local route that the aerial robot or the ground robot can follow in real time. The authors suggest possible algorithm applications, such as warehouse navigation, autonomous driving, courier robots, and scout robots. In [11], the authors proposed a method based on deep learning, a ray tracing algorithm, a waiting rule, and a fast-explorable random tree. The proposed approach uses a neural network to train a model that can predict safe routes for indoor robots. The neural network is trained on a dataset consisting of images of the environment and information about obstacles. The authors consider the robot navigation algorithm in offices, hospitals, etc. The article of [12] describes a new approach to obstacle avoidance using the Floyd–Warshall differential evolution algorithm. It allows the shortest paths between all pairs of points on the graph to be found. The graph represents the environment in which the UAV must avoid obstacles. Firstly, a graph of the environment is built using UAV sensors. After that, the evolutionary algorithm of Floyd–Warshall differential evolution is used to find the shortest route from the current position of the UAV to the target while avoiding obstacles. The study of [13] considers the issue of designing a quadcopter trajectory in the shortest time through a series of defined waypoints, using all the advantages of quadcopter dynamics. Firstly, a graph of the environment is built using data from the sensors of the quadcopter. The A* algorithm is then used to find the shortest route from the current position of the quadcopter to the target while avoiding obstacles. The authors of [14] propose an approach for planning a UAV flight route that bypasses obstacles and connects the start point to the endpoint in a closed space. The approach creates an optimal route using A* algorithms and hill climbing with a delay. The work of [15] considers the issue of planning UAV routes indoors. The research demonstrates the development of an improved method for planning probabilistic roadmaps for safe indoor flights based on the assumption of a quadcopter UAV model. Experiments have confirmed that the method ensures safe indoor UAV flights while significantly increasing computational efficiency. To maximise the information collected by multicopters during the inspection of large buildings, the task of finding an approximately optimal path passing through a series of desired observation points in a three-dimensional environment with obstacles is considered [16]. The proposed method includes stages of global and local route planning. In global planning, the route from the starting point to the endpoint, bypassing obstacles, is laid approximately using the graph method. At the local planning stage, a more detailed route is formed, considering dynamic changes in the environment and the limitations of the multicopter. For this, the method of route planning based on the potential field is employed.

*LiFi communication with UAVs, including privacy and security issues*. Research on using UAVs to provide LiFi communication indoors and outdoors is gaining momentum. The study of [17] presents the development of an interface protocol for a routing protocol unique to indoor flying ad hoc networks (FANETs) that employ LiFi as a communication channel. An FANET is a network of UAVs that communicate without using base stations. An FANET can be used for various purposes, such as environmental monitoring, parcel delivery, and search and rescue. The article [18] is an in-depth study and analysis of Li-Fi, a cutting-edge technology that transmits data at high speeds using visible light as a medium. It offers a more efficient and secure alternative to Wi-Fi and detailed explores relevant research fields and methods. To protect information and data in an FANET, the authors of [19] evaluate the possibility of using LiFi with several UAVs for indoor cooperation and cooperative networks. The works of [20,21,22] consider using LiFi and hybrid WiFi/LiFi technology to overcome traffic restrictions. When describing the theory of LiFi, the latest research in the field is presented. The authors of [23] discuss a new system concept for LiFi in industrial wireless applications that employs a multi-input, multi-output distributed multi-user architecture that provides seamless mobility, reliable low-latency communication, and integration with positioning and 5G. The paper of [24] investigates the hypothesis that LiFi communication methods based on deep learning can effectively learn the characteristics of the indoor environment and user behaviour to perform better than conventional channel estimation methods, mainly when access to state information channels in real time is limited. The authors of [25] propose new realistic channel models based on measurements for internal LiFi systems. In particular, channel gain statistics for randomly oriented fixed and mobile LiFi receivers were obtained. Based on the models obtained, the influence of random orientation and spatial distribution of LiFi users was investigated, showing that the factors above can strongly influence channel gain and system performance. The article of [26] explores the time-varying characteristics of a mobile LiFi channel based on measured data. The resulting rate expression shows that mobile LiFi communication is possible using at least two photodiodes with different orientations. The authors of [26] emphasise that the proposed channel estimation and tracking schemes effectively design mobile LiFi systems. The authors of [27] describe the system architecture for LiFi and 5G in intelligent manufacturing and consider the advantages of using these technologies in developing Industry 4.0/5.0 concepts. The work of [28] considers LiFi technology for building a network using a swarm of UAVs. There is an analytical derivation of the average block error probability using a Chebyshev approximation, lower, and upper bounds. However, the authors of [28] ignore the issue of route planning and the actual deployment of the flying UAV LiFi network in interference conditions. The article of [29] proposes a method of traffic prediction for UAV networks, considering connection errors to access points. The study uses stochastic evolutionary game theory to solve analytically the change in throughput for each connection failure probability. The article of [30] presents two functional test benches for checking the accuracy of real-time light transmission. LiFi technology at different distances and with different angular placements of lighting devices and end receivers with local and Internet connections is evaluated. The authors of [30] emphasise that distance and angular position do not affect LiFi data transmission when downloading and at different distances. The work of [31] considers the issue of a reliable LiFi network within the framework of spatial random locations of terminals in three-dimensional space. Studies are conducted to analyse the performance of a LiFi channel, providing closed-form expressions for the outage probability and average bit error rate for on–off keying under indirect modulation or direct detection at the receiver. The dynamic development of UAVs and LiFi technologies makes it possible to describe in detail the main characteristics of UAVs, which are necessary to solve the problem of signal transmission and reduction. The authors of [32] analyse the performance of the unified physical layer based on neural networks for hybrid LiFi/WiFi networks using UAVs in indoor spaces. Using flying UAVs as network nodes in hybrid LiFi/WiFi networks can provide several benefits, such as improving service quality, increasing flexibility and deployment, and reducing interference and delays. However, the authors also identify possible challenges, such as dynamic changes in network topology and the need to ensure security and privacy. In turn, the authors of [33] present an overview of the use of software-defined networks and the virtualisation of network functions for UAVs. Software-defined networks and the virtualisation of network functions are technologies that increase networks’ flexibility, scalability, and controllability. The article of [34] provides an overview of the use of UAVs to support peripheral computing in 6G Internet of vehicles networks. The authors note that using UAVs to support 6G Internet for vehicles has several advantages, such as reducing delays, increasing productivity, expanding network coverage, improving communication reliability, etc. When reviewing the technology, the authors of [35] consider using LiFi for vehicle communication. It is noted that LiFi has a number of advantages over traditional radio frequency technologies such as Wi-Fi and Bluetooth, including high bandwidth, security, and immunity to interference. These advantages make LiFi an ideal vehicle-to-vehicle communication technology that can be used to improve road safety and transport efficiency. The authors of [36] discuss the progress made in improving the performance of LiFi systems, such as increased bandwidth, range, and security. The main idea is that LiFi is a promising wireless data transmission technology with many advantages over radio frequency technologies. LiFi can be used in 5G, IoT, and cyber security applications. LiFi has the potential to revolutionise the wireless industry by providing higher bandwidth, security, and energy efficiency than traditional RF technologies. However, the technology also has a number of limitations that must be considered when choosing it for a specific task, for example, a limited range and the need for specialised equipment, which can be critical when using UAV swarms. Combining possible data transmission technologies is also a relatively common area of research. The authors of [37] provide an overview of modern and critical research areas in the development of hybrid optical wireless networks. The study examines various combinations of hybrid systems, including RF/optical and optical/optical systems. It is noted that hybrid optical wireless networks are a promising solution to meet the growing demand for high-speed and reliable wireless communication. The article of [38] discusses security issues and possible attacks on the communication channel, such as the following:

- Privacy attacks: since LiFi uses visible light, it is possible to intercept data by analysing light fluctuations;
- Data integrity attacks: attackers can change LiFi data by manipulating the intensity or frequency of light vibrations;
- Availability attacks: attackers can block or disrupt LiFi data transmission by blocking or altering the light signal.

Various LiFi security techniques, such as data encryption, authentication, and attack detection, are also discussed. In general, LiFi is a secure technology, but specific security measures must be taken to protect data and LiFi networks from attacks, such as the following:

- Data encryption to protect LiFi data from interception and further modification;
- Authentication to improve preventing unauthorised access to LiFi networks;
- Development and implementation of attack detection systems that can block and minimise the risks of successful attacks on LiFi networks.

The paper of [39] analyses the secrecy performance of LiFi networks, considering random device orientation and partial knowledge of eavesdroppers’ channel state information, and presents a machine learning-based AP selection algorithm that enhances the secrecy capacity by up to 30%. Since some of the equipment in a flying LiFi network may be Internet of Things (IoT) devices, approaches related to the use of FL-based approaches to enable the collaboration among IoT devices to train machine learning models are of interest [40,41]. The paper of [40] proposes a three-layer federated learning architecture optimised for improving the training efficiency and accuracy in UAV-assisted edge computing for IoT, addressing challenges like user terminal variability, data quality, and dynamic wireless networks. The paper of [41] proposes a three-layer federated learning architecture optimised for improving the training efficiency and accuracy in UAV-assisted edge computing for IoT, addressing challenges like user terminal variability, data quality, and dynamic wireless networks. The authors of [42] present a time-of-flight indoor positioning system for LiFi based on the ITU-T G.9991 recommendation. The proposed positioning algorithm is based on coarse timing using a frame synchronisation preamble, similar to ranging, and fine timing using a channel estimation preamble. Field results show that G.9991-based positioning can achieve an average distance error of several centimetres in 3D. Considering the widespread use of indoor lighting and the availability of an advanced optical wireless communication system using G.9991, the proposed LiFi positioning is a promising new feature that can be added to existing protocols and further expand the capabilities of intelligent lighting systems for the benefit of Industry 4.0. Having support in the form of appropriate software tools is essential for deployment planning.

*Simulation and modelling for UAVs*. The issue of modelling the operation of UAVs is considered in many works. For example, [43] emphasise that modelling is essential for developing and testing UAVs. Simulations allow engineers to study the behaviour of UAVs in different environments, which can help them avoid expensive and dangerous tests on real UAVs. The authors of [44] presented a computer simulator for simulating the behaviour of UAVs in an environment with obstacles. In doing so, they used graph-based planning, feedback-based control, and various machine learning techniques. In [45], the implementation of a ground control station for a UAV flight simulator is considered. The ground control station allows the operator to control the UAV in the simulator and observe its behaviour. In [46], the influence of ammunition launch on the stability of six-rotor UAVs is investigated using computer simulation. The authors analyse the impact of various factors, such as the angle of launch of ammunition, launch time, installation position, and mass, on the stability of the UAV. The article of [47] discusses the design of a ground flight simulator with adjustable stability for pilot training. Such a simulator makes it possible to simulate the behaviour of an aircraft in various flight conditions, including changes in aerodynamic characteristics and failures of control systems. It helps pilots to practise their piloting skills and deal with unexpected situations. The authors of [48] discuss the key software components and functions required for the safe and efficient control of UAVs. The authors of [49] emphasise the importance of choosing a simulator based on scalability, reliability, and ease of use. The work of [50] discusses the UTSim framework, designed to integrate UAVs into air traffic control. The works analysed above on applying LiFi technology were mainly devoted to stationary solutions for closed premises. Recently, flying LiFi networks have been considered fundamentally possible; however, rather exotic technical solutions have become increasingly popular for indoor and outdoor wireless communication. The article of [51] demonstrates the possibilities of using a flying LiFi network to collect and transmit weather data to a ground station for further processing. In [52], the feasibility of using a flying LiFi network indoors to support solutions based on 6G technologies is substantiated.

*Reliability and dependability in UAV swarms*. Since it is essential to ensure its reliable functioning after the deployment of the flying network, it is worth touching on the works that are tangential to this issue. Many works are devoted to ensuring the reliability of UAV swarms (fleets) involved in deploying flying wireless networks. For example, the article of [53] presents a model of the reliability of a UAV swarm that deploys a wireless flying network in intense combat operations based on a continuous-time Markov chain. The work of [54] proposes a new method for assessing the reliability of UAVs’ swarm with different types of redundancy, which enables the important measure for determining the UAVs whose malfunction has the most significant impact on the failure of the flying wireless network. Models for assessing the reliability of a fleet of UAVs with centralised and decentralised control, which are based on the representation of this fleet as a system with binary states, are developed [55]. The algorithms for overflight and providing surveys of given points using a fleet of UAVs are designed [56]. The authors of [57] developed and explored the reliability models of monitoring points for a flying wireless network based on groups of UAV fleets with a centralised, decentralised, and mixed scheme of activation of reserve UAVs and with the possibility of redundancy of control points recommendations were formulated for the selection of redundancy options. In addition, the work of [58] investigated algorithms for planning the placement of UAVs at communication support points in destruction conditions. However, it did not provide algorithms for deploying the flying network and ensuring its reliability and autonomy. Systems of monitoring critical facilities, in particular, nuclear NPP installations, which are based on UAV swarms and operate in pre- and post-accident conditions, are considered in [59,60]. In [60], such monitoring is carried out using Digital Twins technology. The reliability models presented in this paper take into account the sensor coverage of the monitoring zones. However, dependability models do not consider the temporal changes in the reliability and the possibility of replacing drones and the reserve of their autonomy. The tasks of modelling mobile and smart systems considering the complexity of hardware and software components and availability models considering possible cyber-attacks on network and sensor elements are analysed in [61] and [62], respectively. Such models and simulation approaches [62] can be useful for assessing the dependability of flying networks of a given class but must be adapted accordingly. The IT infrastructure of flying networks can be based on IoT technology, particularly on the Internet of Mobile Things [63]. In this case, to assess the dependability and resilience, vulnerabilities of IoT protocols [64] and other cyber risks for UAV swarm assets should be considered [65,66].

Thus, based on the analysis of the references above, we conclude that during the deployment of wireless flying networks on-premises, the following tasks are essential:

- Creation of hybrid LiFi/WiFi networks and their use depending on the complex conditions of closed premises and outside them;
- Laying routes of signal propagation and movement of the UAV inside the room to bypass obstacles using various algorithms of bypassing;
- Ensuring the reliable functioning of flying wireless networks within a given time;
- Development and application of software tools to support the planning of the deployment of flying wireless networks.

Consequently, LiFi networks are becoming increasingly in demand due to indoor wireless deployment issues. Moreover, model and software support are needed to ensure reliable and safe operation in rooms with obstacles.

### 1.3. Objectives and Structure

This research aims to ensure reliable functioning and reduce the deployment time of the flying LiFi network by developing and implementing methods and software tools to support the planning and deployment of UAVs in destruction conditions. The destruction condition of the production facility is understood to be such a state characterised by various obstacles formed due to damage to technological equipment and building structures.

To achieve this goal, the following objectives are designed:

- Analyse the existing methods of planning the deployment of wireless networks in conditions of destruction;
- Develop methods and algorithms for planning the placement and deployment of the UAV flying LiFi network to ensure data transmission in conditions of destruction;
- Develop a method of increasing (ensuring) the reliability of the flying LiFi network, considering the requirements for the reliability and autonomy of UAVs;
- Develop software tools for the support system for planning the UAV flying LiFi network deployment.

The rest of this paper is structured as follows. Section 2 describes the investigation methodology, the main stages and substages, the content and connections between stages, and the research results. This section determines the logic and consequence of solving the tasks presented in Section 3, Section 4 and Section 5. Based on the results of UAV location planning, strategies and algorithms for deploying UAVs as elements of flying networks are developed and explored in Section 3. Section 4 describes methods for ensuring the operational reliability of the flying network that provides dependable communication during the required time. Section 5 presents software tools to simulate and assess the developed algorithms and support decision making at the stages of deployment and operation (ensuring reliability). The results of the investigation are discussed in Section 6. Section 7 briefly summarises the outcomes obtained and identifies future research directions and prospective developments.

## 2. Methodology

Within the framework of this study, the principles of system analysis were used in the development of the formulation and solution of partial research problems, namely the following:

- When decomposing the assigned tasks into stages and determining the logic of their implementation;
- Selection of research methods, mathematical apparatus, and their relationship with the results of individual stages;
- Construction of deployment planning methods, actual deployment, and reliability improvement of the created flying LiFi network in a room with static interference.

**Figure 1:**
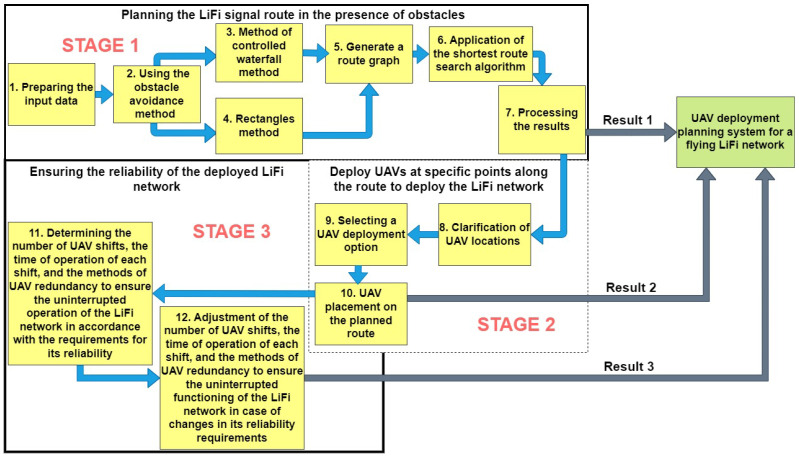
Diagram for deploying a UAV-based LiFi network with obstacles in a production facility.

To solve the formulated scientific and applied tasks, a planning scheme for deploying a flying LiFi network based on UAVs in a production facility with obstacles was developed (Figure 1), according to which such planning takes place in three stages.

**Stage 1.** *Laying the LiFi signal propagation route in conditions of interference*. In the simplest version, the task can be formulated as follows: laying a route (LiFi communication line) from point A (source of information) to point B (consumer of information) in a production facility with obstacles in two-dimensional (2D) space is needed. Although the use of 2D space may limit flexibility in choosing optimal routes, especially in the face of changing environments or navigation accuracy requirements, 2D modelling is also desirable for the following reasons:

- In 2D space, the task of laying LiFi signal propagation routes around obstacles can be more straightforward compared to 3D space and reduce the computational load on the flying LiFi network deployment planning support system, thereby speeding up the decision-making process;
- Modelling scenarios in 2D space can allow the use of simpler and cheaper sensors and navigation systems, reducing the equipment’s cost and complexity.

It is also necessary to accept the assumption that the brightness of the light in the room and the level of its smog (dustiness) allows the use of LiFi technology. In the beginning, if there is an opportunity, it is necessary to assess the consequences of destruction in the production premises and make a map of the obstacles, and for the implementation of the methods discussed further, each of the obstacles will be presented in the form of a convex polygon. Next, we choose one of the methods of bypassing obstacles: the method of rectangles (when bypassing each obstacle is carried out exclusively according to the rule of the left or right corner) or the controlled waterfall method (these methods were developed and discussed in detail in previous works [58,67]). When implementing each method, we assume that points A and B and the obstacles are static and do not change over time. Applying the above methods allows the formation of a set of routes from point A to point B in the 2D space of the production premises. Combining all points of the route (A, B, and points of change in the direction of movement along the route due to bypassing obstacles) generates a graph of routes. The presence of such a graph in the next step allows applying the algorithm for finding the shortest route (for example, Dijkstra’s algorithm) for the propagation of a LiFi signal in conditions of interference from the source of information (point A) to its consumer (point B). The final step of the first stage is processing the obtained results for further use in stages 2 and 3. During the performance of stage 1, methods of system analysis, optimisation, mathematical modelling, and graph theory are used.

**Stage 2.** *Placement of the UAV at the specified points of the laid route for the deployment of the LiFi network*. The first step at this stage is to define a list of movement options for each UAV from its base location to a given route point for the deployment of the LiFi network. In the next step, the best option is selected by the given criterion (for example, the deployment time), and the UAV is placed at the specified points of the laid route. During stage 2, system analysis, mathematical modelling, graph theory, and schedule theory are used.

Thus, the flexible, high-speed, and secure flying LiFi network will be formed after the second stage; such a network will consist of the following:

- Source of information (light-emitting device) encoding data into light signals and transmitting them to the UAVs;
- UAVs serving as mobile repeaters and allowing the LiFi signal to reach areas that are not in direct line of sight of the source of information;
- Consumer of information (a computer, smartphone, or other connected equipment) receiving and decoding the LiFi signal from UAV repeaters.

**Stage 3.** *Ensuring the reliability of the deployed LiFi network*. When the customer provides requirements regarding the minimum required value of the LiFi YBR of the network, the reservation method and the number of reserve UAVs are determined. If the requirements change, the reservation method and/or the number of reserve UAVs are adjusted. During stage 2, system analysis, reliability theory, mathematical modelling, and scheduling theory are used.

The stages’ results can further be used by the flying LiFi network’s UAV deployment planning system, which, in turn, is the main decision-support tool for the person responsible for responding to accidents at critical infrastructure facilities.

## 3. Strategies and Algorithms of Deployment

The deployment stage is based on the results of planning routes, calculation of the required UAV quantity, and their location point for providing communication (see Figure 1). This section describes three various strategies of UAVs routing from the depot to the location points and analyses examples of deployment to support decisions related to the choice of the rational route in point of view of the time and reliability characteristics. The results of this stage are the basis for ensuring operational reliability (Section 4).

### 3.1. Strategies

Since the main goals of this study are to develop UAV deployment strategies for forming a flying LiFi network in rooms with obstacles and to ensure its uninterrupted operation with a certain level of reliability during a given time, the following tasks are to be performed:

- Analysis of various options for placing the UAV on the pre-laid LiFi signal propagation route from the source of information to its consumer in a room with obstacles;
- Development of strategies for deploying UAVs from a stationary depot to form a flying LiFi network;
- Development of methods for ensuring the uninterrupted functioning of the flying LiFi network with the required level of reliability within a given time.

When describing the strategies, the following assumptions were made:

- The propagation route of the LiFi signal (LiFi route) from point A (source of information) to point B (consumer of information), the number of UAVs for the formation of a flying LiFi network, and their placement points on the route are considered as predetermined. A detailed description of the methods of planning the placement of the UAV on the route can be seen here [58];
- The base location of the UAV (depot), which is indicated by point C, does not coincide with points A and B, as well as with any of the UAV placement points on the LiFi route;
- The depot does not change its location over time (it is stationary);
- The number of UAVs is sufficient to cover all designated UAV placement points on the LiFi route;
- UAVs of the same type are used (the term of the same type means with the same characteristics regarding autonomy, speed, failure rate, etc.);
- The time required to deploy the flying LiFi network is defined as the sum of the arrival time of the last UAV from the depot to the designated place on the LiFi route and the time of setting up the flying LiFi network.

UAV deployment strategies to form a flying LiFi network can be divided into two groups:

1. *Individual*. This group of strategies involves the movement of UAVs from the depot to their placement points on the LiFi route along individual routes according to established rules, including obstacle avoidance rules. Such strategies significantly depend on the capacity of the on-board battery of each UAV, since its resource, in addition to ensuring the operation of the flying LiFi network, is additionally spent both on moving to a specified point and on returning to the depot;
2. *Collective*. This strategy involves delivering a group of UAVs to a designated LiFi point of the route using a UAV carrier. The network deployment time criterion can determine the arrival point of the UAV group. These strategies are more complex since an additional entity—a carrier UAV—is introduced into the traffic rules and obstacle avoidance algorithms. However, its use saves the battery resource of each UAV and ensures longer operation of the LiFi network in one deployment.

Next, only a group of individual strategies will be considered, namely the strategy of the first point of the route, the strategy of radial movement, and the strategy of the middle point of the route.

#### 3.1.1. First Point of the Route Strategy

**Figure 2:**
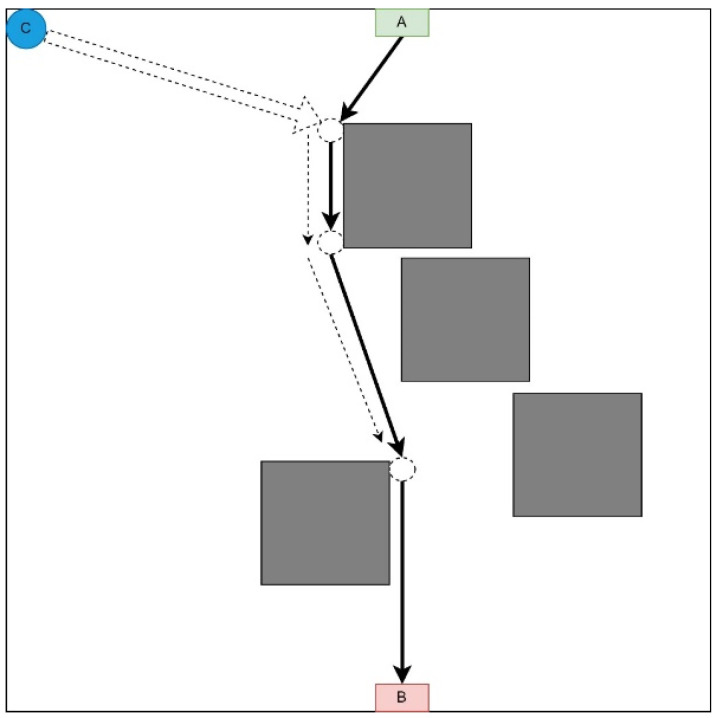
Strategy of the first point of the route representation.

This strategy involves moving each UAV to the first point of the LiFi route and further deploying the network within it (Figure 2). This strategy may be in demand if there are no obstacles on the UAV route from the depot (point C) to the first LiFi point of the route and a significant number of obstacles on the UAV routes to its other points (it is necessary to lay additional routes to bypass these obstacles).

Arriving at the first point, each UAV moves along the LiFi route to the destination point (the location determined for it as part of the deployed LiFi network).

The sequence of departures of UAVs from the depot (point C) is established by decreasing the distance between the depot and the destination point: the UAV furthest from its destination point takes off first, and so on. This strategy is characterised by the significant consumption of the UAV’s on-board battery resources, which is the most distant from its destination.

Table 1 shows data characterising the deployment process of the flying LiFi network according to the strategy of the first point of the route at UAV speeds of 2 m/s, 3 m/s, and 4 m/s.

Thus, with a strategy of the first point, a flying LiFi network of five UAVs at a UAV speed of 2 m/s can be deployed in 41.38 s, with a speed of 3 m/s in 38 s, and with a speed of 4 m/s, it can be deployed in 36.6 s.

#### 3.1.2. Radial Movement Strategy

**Figure 3:**
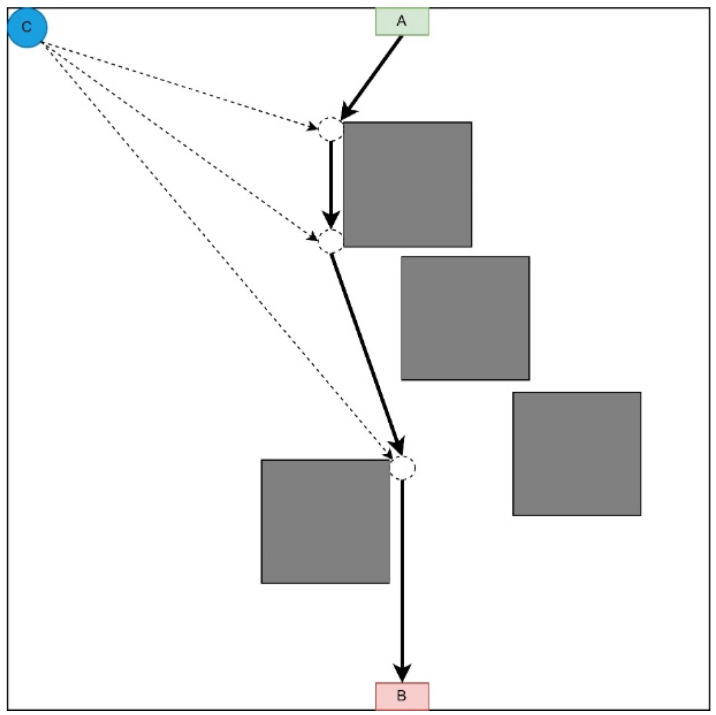
Radial movement strategy representation.

This strategy involves moving each UAV immediately to the destination point on the LiFi route (Figure 3).

It is advisable to use this strategy if there are no obstacles on most UAVs’ movement routes from the depot to the destination point. This strategy prevents queuing at the LiFi points along the route as each UAV follows its route to its destination. The order of departure of UAVs from the depot also occurs in the order of decreasing distance between the depot and the destination point.

Table 2 shows data characterising the deployment process of the flying LiFi network using the radial movement strategy at UAV speeds of 2 m/s, 3 m/s, and 4 m/s.

Thus, with a radial movement strategy, a flying LiFi network of five UAVs at a UAV speed of 2 m/s can be deployed in 41.38 s, with a speed of 3 m/s in 37.59 s, and with a speed of 4 m/s in 35.69 s.

#### 3.1.3. Midpoint Strategy

**Figure 4:**
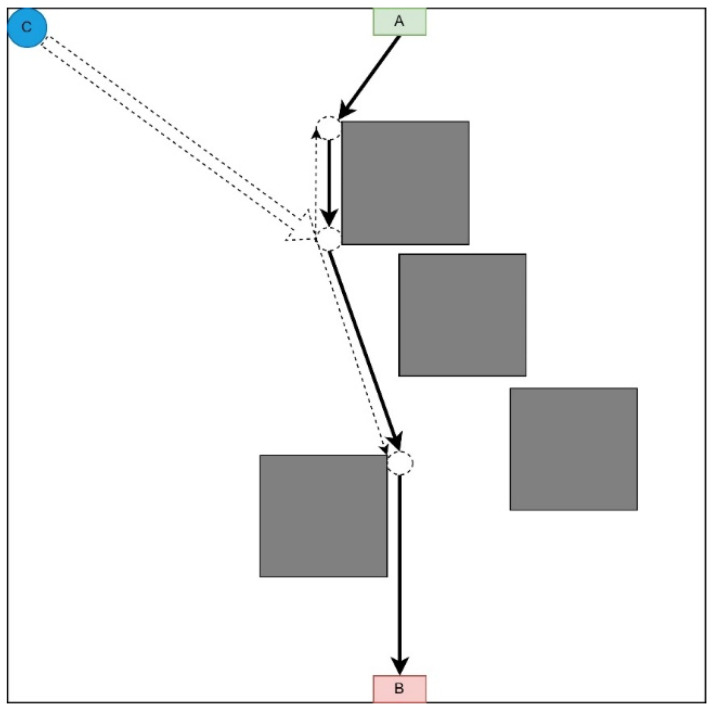
Midpoint strategy representation.

This strategy involves moving each UAV to a point as close as possible to the middle of the LiFi route and further deploying the network within it (Figure 4).

This strategy is appropriate if there are no obstacles on the UAV route from the depot to the corresponding LiFi point of the route and there are many obstacles on the UAV routes to its other points (it is necessary to lay additional routes to bypass these obstacles).

Arriving as close as possible to the middle of the LiFi route, each UAV moves along the LiFi route in the direction of the destination—either in the direction of point A or in the direction of point B. The sequence of UAV departures from the depot is in the order of decreasing distance between the depots (point C) and the destination point. Movement in both directions of the LiFi route will reduce the likelihood of queues at this point.

Table 3 shows data characterising the deployment process of the flying LiFi network according to the strategy of the midpoint of the route at UAV speeds of 2 m/s, 3 m/s, and 4 m/s, respectively.

Thus, with a midpoint strategy, a flying LiFi network of five UAVs at a UAV speed of 2 m/s can be deployed at 41.61 s, with a speed of 3 m/s in 37.74 s, and with a speed of 4 m/s, it can be deployed at 35.81 s.

**Figure 5:**
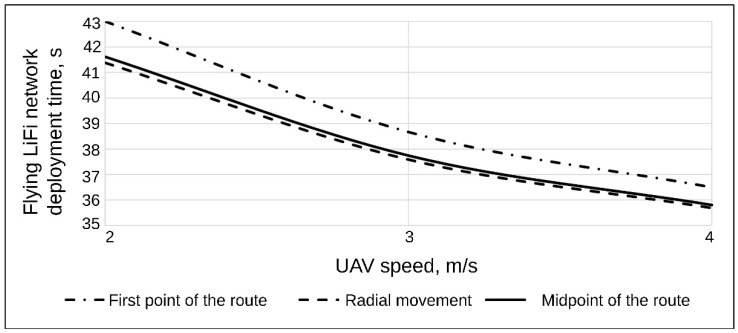
The graph shows the dependence of the flying LiFi network’s deployment time on the UAV’s speed for the strategies of the first point of the route, radial movement, and the route’s midpoint.

For a comparative analysis, Figure 5 shows a graph of the dependence of the flying LiFi network’s deployment time on the UAV’s speed for each strategy.

The graph analysis in Figure 5 yields significant insights into the deployment time of the flying LiFi network, particularly concerning the speed of the UAV and the chosen strategies.

1. The most substantial reduction in the deployment time of the flying LiFi network, resulting from the increase in UAV speed from 2 m/s to 4 m/s, is particularly noteworthy. This reduction is observed for the strategy of the first point of the route, which is 6.5 s. The strategies for the radial movement and the midpoint of the route are 5.69 s and 5.81 s, respectively. When ranking the strategy based on the increasing gain in time from the UAV speed increase, the sequence is as follows: the radial movement strategy, the strategy of the midpoint of the route, and the strategy of the first point of the route.
2. For all considered UAV speeds, the radial movement strategy ensures the shortest deployment time of the flying LiFi network. For example, for a speed of 2 m/s, this time is 1.39 s less than the deployment time of a flying LiFi network when applying the strategy of the first point of the route. Suppose we rank the strategies to decrease the deployment time of the flying LiFi network. In that case, the result will be the following sequence: the strategy of the first point of the route, the strategy of the midpoint of the route, and the strategy of the radial movement.

Considering the research results, Table 4 summarises the advantages and disadvantages of each strategy.

### 3.2. Application Examples

**Figure 6:**
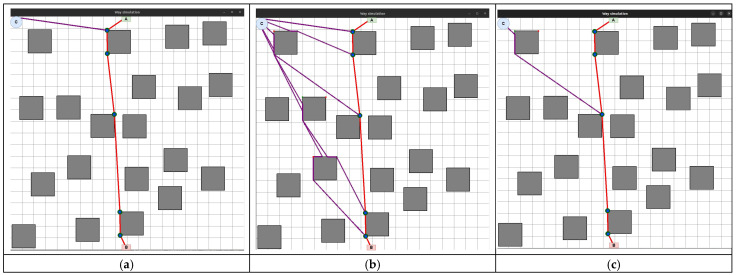
The flying LiFi network is deployed using the following strategies: (**a**) the first point of the route; (**b**) radial movement; (**c**) midpoint.

The development of the UAV deployment method according to the strategy of the first point of the route was preceded by the laying of the LiFi route and the marking of UAV placement points on it for the formation of a flying LiFi network using the controlled waterfall algorithm to bypass obstacles. The simulation was carried out using the developed “Simulation Way” software tool (a detailed description of the tool is given in Section 5 of the paper). The simulation results are illustrated in Figure 6a, which shows the following:

- The working area of the production premises with 20 rectangular obstacles measuring 2 × 2 m each;
- Route laid to bypass LiFi obstacles (shown in red);
- UAV placement points on the LiFi route to form a flying LiFi network (shown by green dots). These points were obtained taking into account the limitation on the maximum possible distance between UAVs (its increase may exceed the maximum possible range of the LiFi signal set for the given conditions);
- Source of information (indicated by a green rectangle with the letter A inside);
- Consumer of information (indicated by a red rectangle with the letter B inside);
- Depot for UAVs (marked by a blue circle with the letter C inside).

Next, a first point strategy was applied to deploy the flying LiFi network. The modelling results using the “Simulation Way” software tool are also illustrated in Figure 6a, where individual UAV movement routes to their destinations on the LiFi route are shown in blue.

The same LiFi route according to the managed waterfall method was used to deploy the UAV according to the radial movement strategy, and a radial movement strategy was used to deploy the flying LiFi network.

The modelling results using the “Simulation Way” software tool are illustrated in Figure 6b, where individual UAV movement routes to their destinations on the LiFi route are shown in blue. According to the controlled waterfall algorithm, obstacles were avoided during the construction of UAV traffic routes from the depot to the destination points.

The midpoint strategy has used the same approach (see Figure 6c).

## 4. Methods for Ensuring Operational Reliability

According to the described methodology (Figure 1), operational reliability is ensured after solving UAV deployment tasks and choosing routes from the depot to the location points (Stage 2). This section presents developed approaches, models, and algorithms for ensuring flying network reliability, considering the schedule of the main and redundant UAVs. These algorithms are based on the various network deployment strategies developed in Section 3.

### 4.1. Approach

One of the requirements for a deployed flying LiFi network can be as follows: to ensure the continuous operation of the network with the probability of failure-free operation (PFFO) P(t) should not be lower than the minimum acceptable value $P_{min}$ during a given time. This can be, for example, the time of transmission to the consumer of information from sensors about the condition of the equipment, the level of atmospheric pollution in the room, a video stream that gives an idea of the degree of damage in the room, or the presence of injured persons in it.

When considering the methods of ensuring the reliability of the flying LiFi network by the specified requirement, we will accept the following assumptions:

- All UAVs are of the same type and are characterised by the same failure rates λ, 1/hour;
- To ensure uninterrupted operation of the flying LiFi network, two shifts of UAVs are used with the same number of UAVs in each of them n, and this number is equal to the number of their placement points on the laid LiFi route;
- A working UAV shift must be replaced by another shift at the latest $t_{crit}$ of achievement by the working shift of the minimum permissible value of PFFO $P_{min}$ even with the availability of UAV battery life $t_{batt}$, that is sufficient to continue their operation as part of the flying LiFi network;
- Each of the shifts before the start of the first cycle of its work is characterised by PFFO $P_{0}=1$;
- From each subsequent work cycle, the shift begins its work with the PFFO that it reached at the time of returning to the depot;
- After returning to the depot, the battery life is renewed.

### 4.2. Models

Planning departures of changes for the formation of a flying LiFi network considers the fact that each of these changes can be in two states—the state of functioning as part of the LiFi network, the duration of which is calculated according to Formula (1), and the waiting state, in which the change is over time $t_{wait}$.

(1) $$t_{funct}=t_{set}+t_{transm}$$

where

$t_{set}$—time to set up the flying LiFi network;

$t_{transm}$—operation time of the flying LiFi network in data transmission mode.

**Figure 7:**
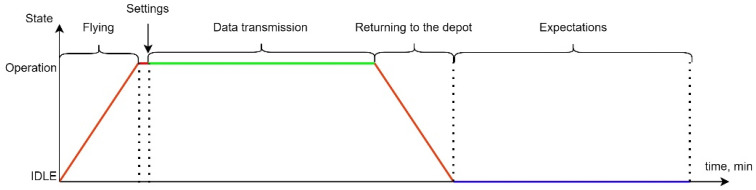
A one-shift work cycle is used to deploy and ensure LiFi functionality.

The transition from the standby state to the operating state continues during the flight time of the UAV from the depot to its points on the LiFi route $t_{arriv}$, and from the state of operation to the state of standby—during the return time of the UAV from the LiFi route to the deport $t_{return}$. Thus, the complete work cycle of a shift can have the form presented in Figure 7, and its duration will be calculated according to the following formula:

(2) $$t_{shift}={t_{arriv}+t}_{set}+t_{transm}+t_{return};\;{t_{batt}>t}_{shift}$$

The PFFO of each shift at the time of return to the depot in the first cycle of work can be calculated with the Formula (2), and in the second and subsequent cycles (k=2,…,m) with Formula (3):

(3) $$P_{1}\left(t_{shift}\right)={P_{0}\text{×}e}^{-n\lambda t_{shift}};\;P_{1}\left(t_{shift}\right)>P_{min}$$

(4) $$P_{k}\left(t_{shift}\right)={P_{k-1}\times e}^{-n\lambda t_{shift}};\;P_{k}\left(t_{shift}\right)\text{>}P_{min}$$

Thus, each shift can work for no more time $t_{crit\_1}$ (Formula (5)) in the first cycle and time $t_{crit\_k}$ (Formula (6)) in the second and subsequent cycles:

(5) $$t_{crit\_1}=\frac{\ln\left(\frac{P_{0}}{P_{min}}\right)}{n\lambda }$$

(6) $$t_{crit\_k}=\frac{\ln\left(\frac{P_{k-1}}{P_{min}}\right)}{n\lambda }$$

**Figure 8:**
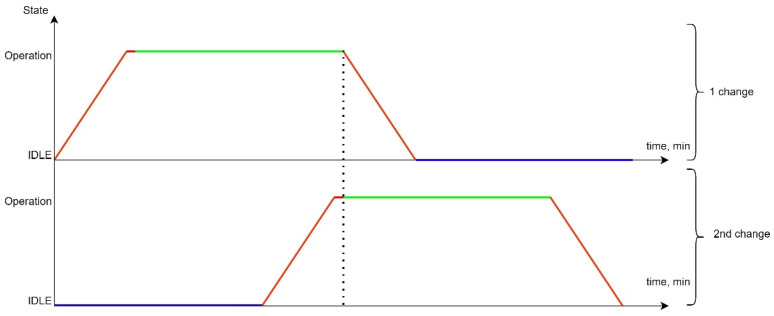
Alternate work of two shifts to deploy and ensure the functioning of the flying LiFi network.

To ensure the continuity of data transmission, it is necessary to ensure the departure of the next shift on ${{(t}_{arriv}+t}_{set})$ ahead of time $\left(t_{shift}-t_{return}\right)$, which is shown in Figure 8.

The following colouring scheme was used on the Figure 7 and Figure 8:

- The red colour indicates the transition process of the current UAV queue from one functional state to another. In our case, it is either the process of UAV flight from the depot to the point on the route, or the process of flight from the point on the route to the depot after the end of the queue. In summary, the red line is the state of the network when the UAVs are in use, the LiFi network in the process of deployment. The probability of failure-free operation in this state varies;
- The green colour indicates the process of supporting an active LiFi network. The probability of fail-safe operation in this state varies;
- The violet colour characterizes the status of the UAV waiting in the Depot/preparation for the next departure. The probability of fault-free operation in this state does not change.

The next step of the research was developing software tools for modelling. These tools were used to ensure the continuous operation of the network with a PFFO not lower than the minimum permissible value during the given time by using two shifts of *n* UAVs each.

### 4.3. Examples

Consider the case when the number of UAVs in each shift, according to the previously accepted assumptions, equals the number of points on the LiFi route, which is 6 (n = 6 UAVs). Let the following input data be given: UAV failure rate. λ = 0.0005 1/hour; $P_{0}$ = 1; $P_{min}$ = 0.99875.

**Figure 9:**
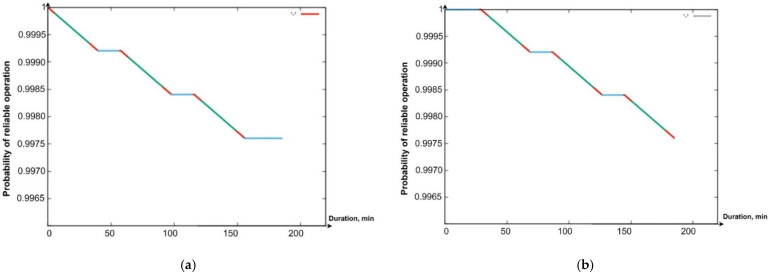
Graph of the dependence of the probability of fault-free operation of the (**a**) first shift in time; (**b**) second shift in time.

**Figure 10:**
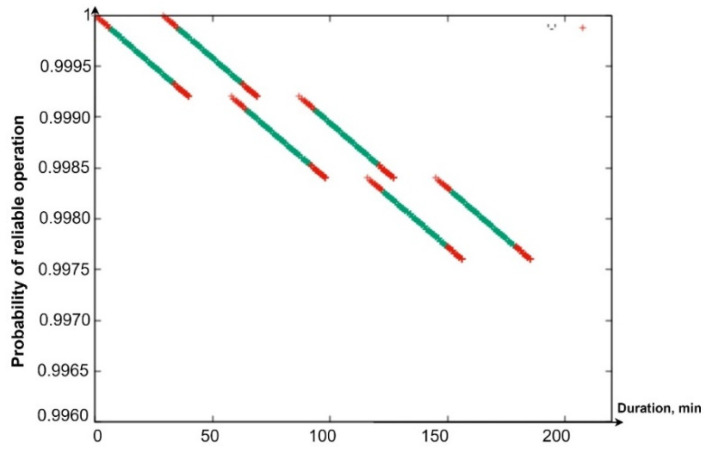
A general graph of the dependence of the probability of PFFO on time for both shifts.

Using the software tool “Reliability Level” (software integrated in “Simulation Way”. The detailed description will be given in Section 5 of the paper), in particular, the Gnuplot library integrated into it, graphs were drawn of the reduction in the PFFO of the flying LiFi network to the minimum permissible value when alternately ensuring the functioning of the flying LiFi network for the first and the second (Figure 9a,b) and two shifts (Figure 10) at the same time.

The coloured sections of the graphs have the following meaning:

- Segments in red show a decrease in the PFFO both during the flight of the UAV to its points on the LiFi route and the setting up of the flying LiFi network (upper red segments) and during the return of the UAV to the depot (red segments);
- Segments of green colour illustrate the reduction in the PFFO during the time of data transmission from the source to the data consumer;
- Blue segments (Figure 9a,b) show the PFFO while waiting for the UAV to change to its next operation cycle.

Analysis of the graphs presented in Figure 9 and Figure 10 allows us to draw the following conclusions:

- The most significant decrease in the FFO is observed during data transmission;
- While waiting for the UAV to change to its next cycle of operation, its PFFO remains unchanged;
- Uninterrupted functioning of the flying LiFi network with the required level of reliability for 180 min can be ensured by two shifts, each of which consists of six UAVs and performs three work cycles;
- The latter ensures the functioning of the flying LiFi network by the second shift in the third cycle of its work.

## 5. Developed Software Tools

Section 2, Section 3 and Section 4 have been dedicated to developing the general methodology, strategies, models, and algorithms for deploying and ensuring the operational reliability of flying networks (see Figure 1, Stages 2–3). Software tools to support decision making in real time are needed to apply these theoretical results. This section describes the tool’s architecture, interfaces, and application examples.

### 5.1. Architecture

The proposed software tool called “Simulation Way” was used to implement the planning stages of the deployment of the UAV flying LiFi network, in particular for the following activities: (i) laying LiFi routes from the source to the consumer of information in a room with obstacles, marking on the routes the points (places) of UAV placement for the formation of a flying LiFi network using left- and right-corner algorithms, as well as a controlled waterfall, to bypass obstacles; (ii) forming a graph of possible LiFi routes and determining the shortest of them by applying Dijkstra’s algorithm; and (iii) placement of UAVs at specified points (places) on the shortest laid LiFi route using different deployment strategies: the first point of the route, radial movement, and the middle point of the route.

**Figure 11:**
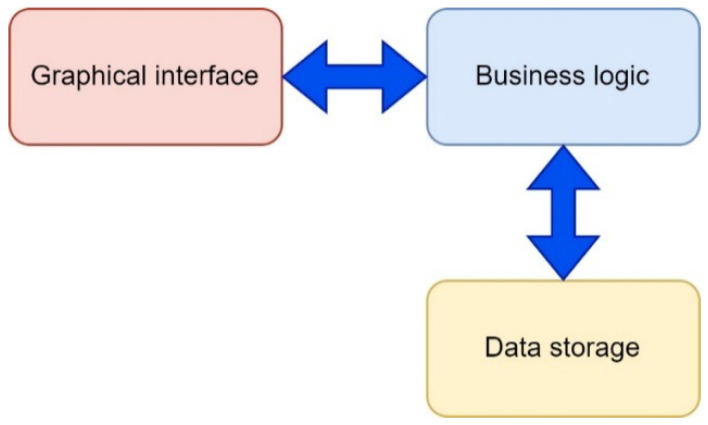
Architecture of the “Simulation Way” software tool.

The architecture of the software tool is shown in Figure 11. The “Simulation Way” software tool has a three-level architecture and consists of the following levels: (i) level of the graphical user interface (GUI). This level represents the interface of the software tool, which is implemented using the Python library called *tkinter*; (ii) business logic level. This level provides the interaction logic between the data storage (storage data) and the graphical interface (GUI). The layer contains modules for generating reports (results), modules for the calculation core, and modules for external algorithms (for example, Dijkstra’s algorithm for finding the shortest route), which can interact with each other.

**Figure 12:**
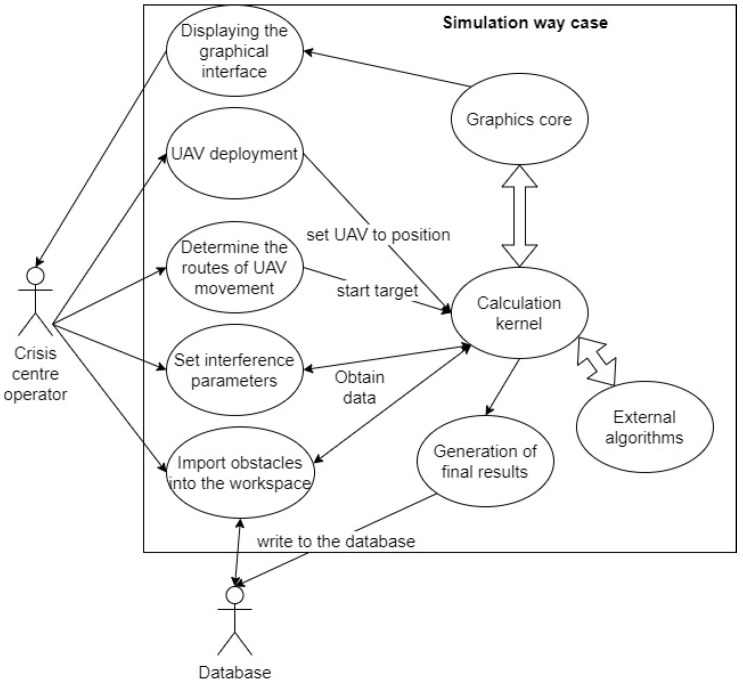
Use-case diagram for the simulation in the “Simulation Way”.

The software tool can be used by CC operators when simulating the deployment of a UAV-based LiFi network in a production facility with obstacles. Options for using the software tool by the CC operator are shown in Figure 12. The interaction of the CC operator with the software tool is carried out using a graphical interface. A large number of settings are available to the CC operator: set the dimensions of the working area of the production premises, generate the required number of obstacles with given characteristics, and choose methods (rules) for bypassing these obstacles. In addition, input data generated during one simulation iteration can be used in subsequent iterations.

The calculation core carries out calculations, combining mathematical algorithmic and interaction modules. The graphic core informs the CC operator about the progress of the modelling process, displays the results of calculations, and allows the modelling rules to be managed. In particular, the CC operator can visually see the following: (i) the iteration number; (ii) the name of the algorithm according to which obstacles are bypassed; (iii) the coordinates of points (places) of UAV placement on the laid LiFi route; (iv) the length of the LiFi route (the length of the given section of the LiFi route); and (v) the number of UAVs that need to be placed at the designated points of the LiFi route for the deployment of the LiFi network.

**Figure 13:**
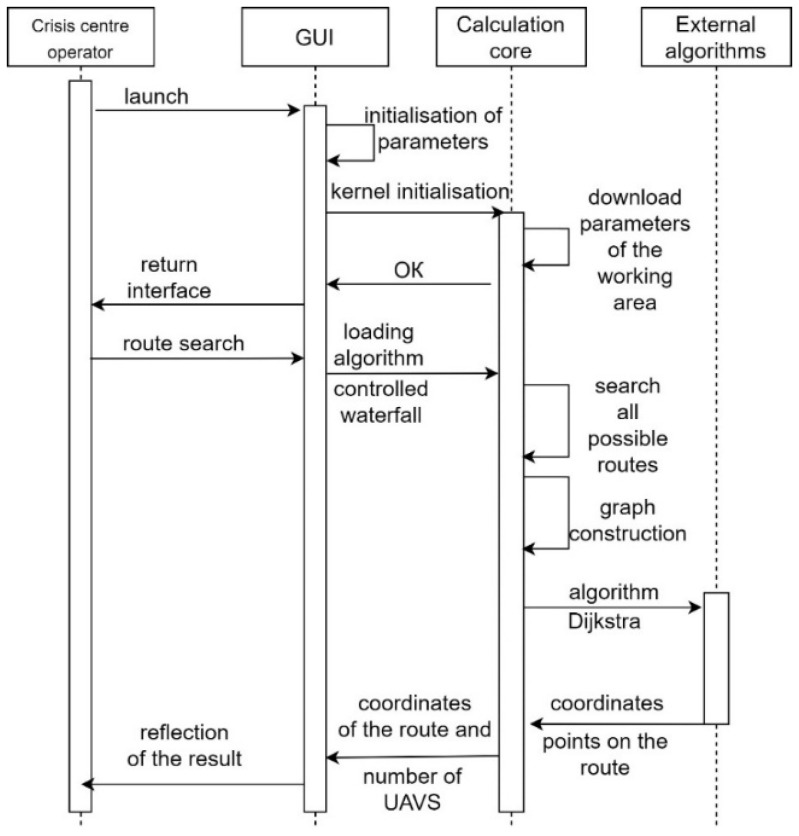
Sequence diagram of the interaction of CC operator with “Simulation Way”.

Figure 13 shows the sequence of the CC operator’s interaction with the “Simulation Way” software tool.

The necessary libraries and modules are initialised during the activation of the “Simulation Way” software tool. The CC operator does not directly interact with algorithms or the calculation core. For this, the CC operator has a graphical interface at his disposal. The functions of the CC operator when using the “Simulation Way” software are to enter (adjust) the parameters necessary for simulation. Based on the LiFi routes generated during simulation, the “Simulation Way” software tool forms a graph of possible LiFi routes. It determines the shortest by applying the Dijkstra algorithm. The receipt of calculation results is possible in the form of visual information on the control panel of the graphical interface and the form of a prepared report file.

CC operators can use the “Reliability Level” software tool to determine the time of operation of the flying LiFi network with a reliability level not less than the minimum permissible during the given time. The console interface allows the CC operator to interact with the “Reliability Level” software tool.

The CC operator can enter the following parameters: (i) UAV failure rates; (ii) setup time of the flying LiFi network; (iii) operation time of the flying LiFi network in data transmission mode; (iv) time resource of the on-board UAV battery; and (v) the minimum acceptable level of reliability with which a flying LiFi network can operate.

### 5.2. Modules and Interfaces

**Figure 14:**
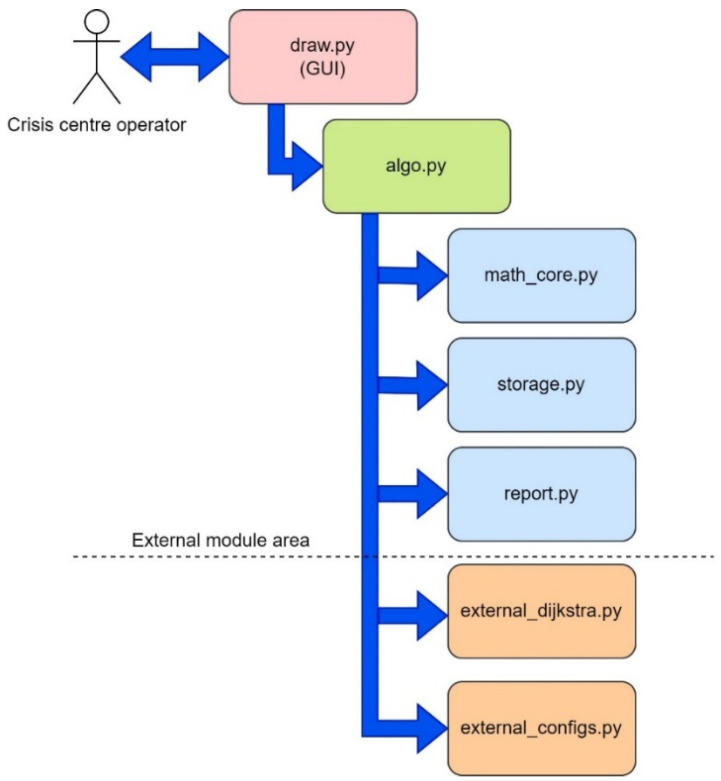
A modular diagram that demonstrates the logic of interaction between the components of the “Simulation Way” software tool.

The “Simulation Way” software tool has a modular structure. Each module implements an API for interaction, separating individual functional blocks. Figure 14 shows a modular diagram demonstrating the interaction logic between the software tool’s components.

The CC operator interacts with the software using the draw.py module. A graphical user interface (GUI) is implemented using *tkinter* (Python library), one of the most common GUI display solutions. The *algo.py* block implements inter-module interaction (obstacle avoidance, UAV movement, etc.) and provides basic functionality. The *math_core* module is the mathematical core of the software. This module implements an API for calculating lengths, converting and correcting floating point numbers, etc. *The external zone* is a zone of external modules that interact with operating system resources and basic integrated algorithms. It is thanks to the external zone that it becomes possible (i) to generate graph images when using various obstacle avoidance algorithms; (ii) to generate data on obstacle parameters, number of iterations, route length (route sections), number of UAVs required for LiFi network deployment, and coordinates of the location point of each UAV on the laid route; and (iii) keep a log of the “Simulation Way” software.

**Figure 15:**
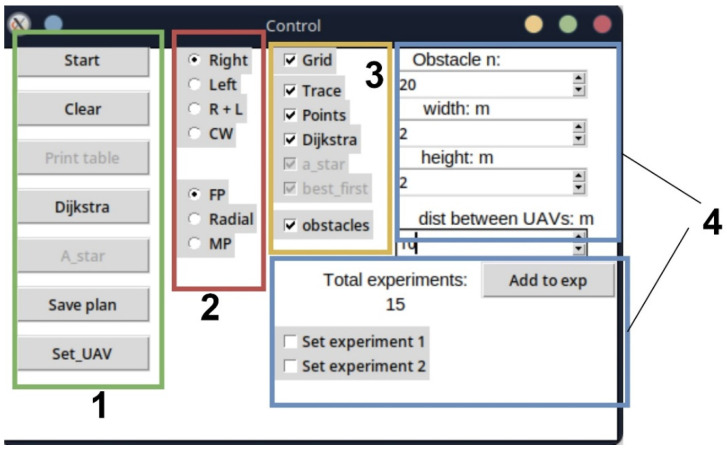
View of the Control panel with functional zones: 1—process launch zone; 2—zone of implementation of the method (rules) of avoiding obstacles; 3—zone of the graphic display of layers; 4—modelling parameters setting area.

**Figure 16:**
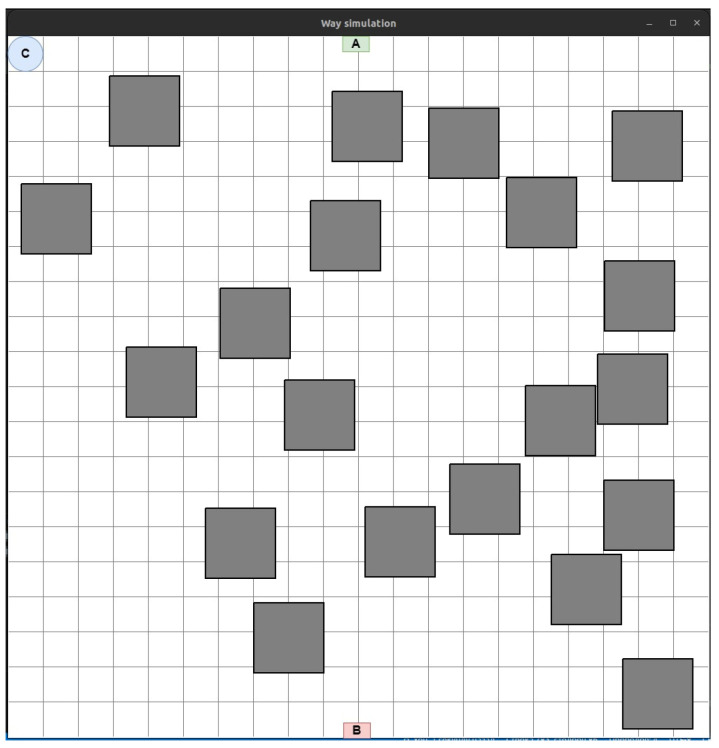
An example of displaying the working area of a production facility with obstacles in 2D space.

The *storage.py* module is a repository for variables required for inter-module interaction. This module stores the characteristics of UAVs, the coordinates of their base locations and further placement points on the LiFi route, the coordinates of the location of obstacles in the room, etc. The data generated using the “Simulation Way” software tool are primarily stored in this module (e.g., the coordinates of the route points for a specific iteration and the name of the obstacle avoidance algorithm). The “Simulation Way” software tool’s graphical interface is located in separate graphic windows: *Control*, which is the control centre for parameters and simulation rules (Figure 15), and *Way simulation* (Figure 16), which displays the progress of the process simulation in real time.

The interface window, shown in Figure 15 can be conditionally divided into four zones.

*Process launch zone.* The Start button is responsible for starting the route search process between the start and end points according to the specified parameters. The Clear button allows data from previous iterations to be cleared. The *Dijkstra* button will enable the use of Dijkstra’s algorithm to find the shortest route in the current graph. *The Start* button combined with the CW item from zone 2 also uses this algorithm, but the graph is generated automatically and cannot be changed during all simulation iterations. *The Save plan* button saves the current room plan and obstacles. The data will be saved in an XML file and can be reused in the future. The *Set_UAV* button is responsible for placing the UAV at the specified points on the laid route.

*Zone of implementation of the obstacle avoidance algorithm*. This zone implements the obstacle avoidance algorithm in the production room. If the Right is selected, obstacle avoidance will be according to the algorithm of the right corner and the Left—the left corner. Selecting R + L will allow simultaneous use of left- and right-corner algorithms. Selecting *CW* will allow the controlled waterfall algorithm to be applied.

*This is a zone for the graphic display of layers*. This zone allows each layer to be turned on and off without data loss or limitations.

*Zone for setting simulation parameters*. This zone allows for setting the number of obstacles and their shape and automates the process of creating statistical data in the report generated for the CC operator.

*The Way simulation panel* (Figure 16) displays the working area of the production room in 2D space.

The small green (marked with the letter A) and red (marked with the letter A) rectangles in Figure 16 are, respectively, the initial (source of information) and final (consumer of information) points of the route. The blue circle (marked with the letter C) shows the stationary depot where the UAVs are located, which will then need to be placed on the laid LiFi route.

In the working area, generated obstacles are depicted as rectangles (in the presence of more complex forms of obstacles, their projection can be inscribed in a convex polygon with an arbitrary number of corners).

### 5.3. Case Study

**Figure 17:**
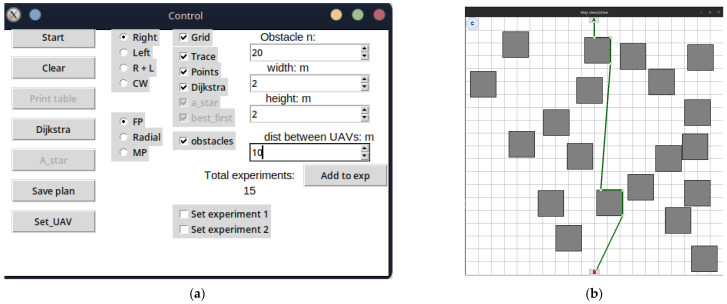
“Simulation Way” tool results for the right-angle algorithm to bypass obstacles: (**a**) control panel with parameters for laying a LiFi route; (**b**) image of a LiFi route.

In this subsection, examples of the use of the “Simulation Way” software tool for laying the route of LiFi signal propagation using the left- and right-corner algorithms, as well as the controlled waterfall algorithm, which are described in detail in the work of [58], will be sequentially considered. To use the correct corner algorithm to bypass obstacles, set the switch to the Right position on the Control panel and press Start (Figure 17a).

The modelling results are presented in Figure 17a. The green broken line shown in this figure is the route laid. In addition, the software tool “Simulation Way” determines and stores the coordinates of points that will be used as UAV placement points as part of the LiFi network formed by them. All vertices of the broken (route) in Figure 17b are considered such points, except for the initial A (source of information) and the final B (consumer of information).

**Figure 18:**
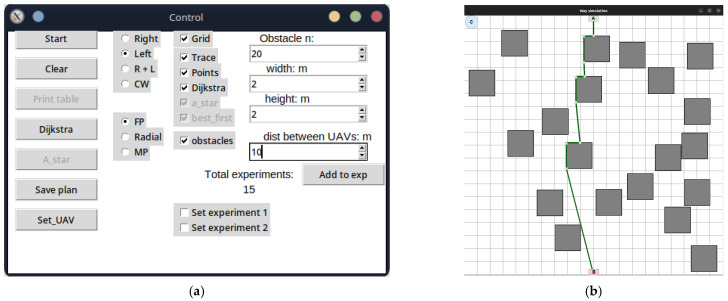
“Simulation Way” tool results for the left-angle algorithm to bypass obstacles: (**a**) control panel with parameters for laying a LiFi route; (**b**) image of a LiFi route.

To activate the modelling process using the left-angle algorithm to bypass obstacles, set the switch to the Left position on the Control panel and press Start (Figure 18a).

As in the previous case, the simulation result will be a generated LiFi route in the form of a green broken line (Figure 18a). To activate the modelling process using the controlled waterfall algorithm to bypass obstacles, it is necessary to set the switch on the Control panel to the CW position and press Start (Figure 18b).

**Figure 19:**
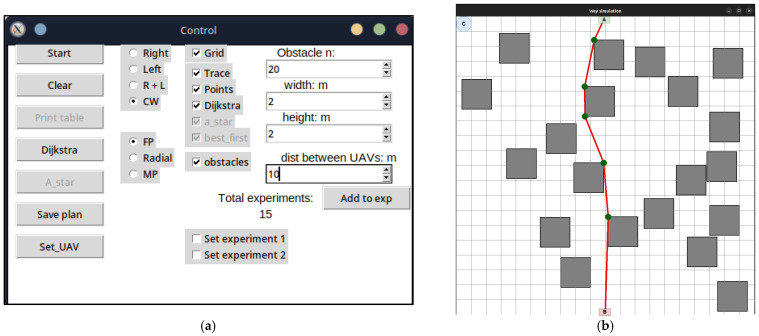
“Simulation Way” tool results for the controlled waterfall algorithm to bypass obstacles: (**a**) control panel with parameters for laying a LiFi route; (**b**) image of a LiFi route.

The laid LiFi route will be displayed as a red broken line with green dots, indicating the UAVs’ locations on the route (Figure 19a). As can be seen, not only the vertices of the broken line but also a certain number of additional points will be the points of future placement of the UAV for network formation. Their inclusion is necessary because, in conditions of increased brightness (dusty, smoky) of the production premises, the distance between the peaks of the broken line may exceed the range of the LiFi signal set for the given conditions. Thus, an additional UAV(-s) must be placed between the UAVs on the points of the adjacent vertices of the broken lines.

To generate the UAV movement routes from the base locations (depots) to the placement points on the laid track, one should press the *Set_UAV* button on the Control panel. The same algorithms can be used to plot the UAV movement routes from the depot to their locations on the laid LiFi route as for laying the LiFi route. In the example presented in Figure 19b, the left- and right-corner algorithms are used simultaneously to bypass obstacles (the switch is set to the R + L position).

**Figure 20:**
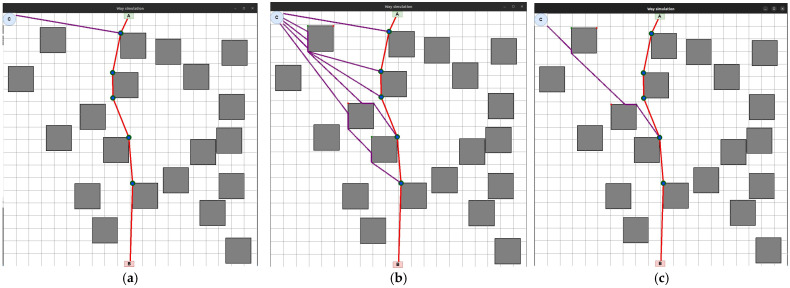
“Simulation Way” results: UAV movement routes from the depot to the placement points (places) on the laid LiFi route using (**a**) the first point of the route strategy, (**b**) the radial movement strategy, and (**c**) the midpoint strategy.

In Figure 20a–c, purple lines indicate the movement routes of each UAV to its location on the laid LiFi route according to the strategies of the first point of the route, radial movement, and the middle point of the route, respectively.

The “Reliability Level” software tool can be used independently or in combination with the “Simulation Way”.

**Figure 21:**
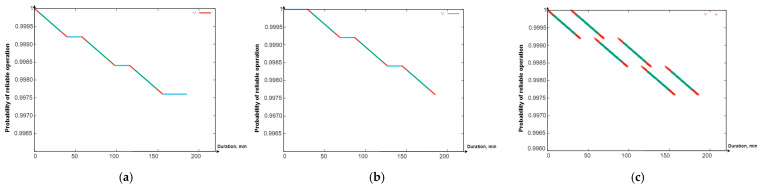
Graphs of the dependence of the probability of fault-free operation: (**a**) PFFO and the first shift on time; (**b**) PFFO and the second shift on time; (**c**) a general graph of the probability of PFFO versus time for both changes.

All data are specified through a configuration file. The work’s results are the graphs presented in Figure 21a—PFFO dependence on time for the first shift, Figure 21b—PFFO dependence on time for the second shift, and Figure 21c—PFFO dependence on time for two shifts simultaneously.

The following colouring scheme was used on the Figure 21:

- Red: part of the graph of the dependence of the probability of FFO on time, which characterizes the time of the transition process (flight from the depot to points on the route, return to the depot, network setup). That is, the section of the route on the graph is marked in red when the UAVs are functioning (their value of the probability of a robot failing) is changing, but they are not yet performing a useful action (the LiFi network is not deployed);
- Green: part of the graph of the change in the probability of FFO over time, which characterizes the actual support of the deployed LiFi network;
- Turquoise: part of the graph of the dependence of the probability of FFO on time, which characterizes the presence of a queue of UAVs in the depot.

The obtained graphs (Figure 21a–c) illustrate the simulation results and calculations, which have a clear physical interpretation, as they describe the change in reliability indicators depending on time and consider all the elements of the work schedule for the main and reserve UAVs. Based on them, depending on (i) the requirements for the reliability and strict continuity of communications and (ii) the spatial location of information sources and receivers, as well as the depot, which affects the time of replacement of UAVs, it is possible to make decisions regarding the choice of UAVs based on the indicators of reliability and reserve of autonomy, their quantity, and replacement procedures.

## 6. Discussion

Based on a comparative analysis of the deployment strategies of flying networks, the following was found:

- The most substantial reduction in the deployment time of the flying LiFi network, resulting from the increase in the UAV speed from 2 m/s to 4 m/s, is particularly noteworthy. This reduction is observed for the strategy of the first point of the route, which is 6.5 s. The strategies for the radial movement and the midpoint of the route are 5.69 s and 5.81 s, respectively. When ranking the strategy based on the increasing gain in time from the UAV speed increase, the sequence is as follows: the radial movement strategy, the strategy of the midpoint of the route, and the strategy of the first point of the route;
- For all considered UAV speeds, the radial movement strategy ensures the shortest deployment time of the flying LiFi network. For example, for a speed of 2 m/s, this time is 1.39 s less than the deployment time of a flying LiFi network when applying the strategy of the first point of the route. Suppose the strategies are ranked in order of decreasing network deployment time. In that case, the result will be the following sequence: the strategy of the first point of the route, the strategy of the midpoint of the route, and the strategy of the radial movement.

It was shown that the work cycle of one shift of a UAV swarm for deploying and ensuring the functioning of the LiFi network consists of the following stages: departure from the depot and approach to specified points on the laid LiFi route; network settings; data transfer; return to the depot; and waiting for the next work cycle. This work does not consider the reconnaissance stage and identification of the general location, number, and types of obstacles. Assumptions about the shape of obstacles are not fundamental.

A graphical interpretation of the alternating work of two UAV shifts to deploy and ensure uninterrupted functioning of the flying LiFi network is provided, according to which the UAVs of the next shift arrive and configure the network formed by them before the UAV of the current shift starts moving to the depot. It should be noted that the proposed two-shift substitution procedure is the simplest. On this basis, developing many other replacement procedures and strategies with whole and fractional multiples is possible, using unique cargo UAVs to deliver changes, etc.

Using the developed software tools, it was calculated that two shifts, each of which performs three work cycles and consists of six UAVs, can ensure the uninterrupted functioning of the flying LiFi network with a PFFO not lower than $P_{min}$ = 0.99875 for 180 min. The shifts have a failure rate of λ = 0.0005 1/hour.

This study develops the results presented in [59], where methods and algorithms for planning the placement of UAVs were developed to ensure continuous Li-Fi communications in destruction conditions. The task of deploying and ensuring the reliability of the flying network is a natural continuation of the study from the point of view of the entire operational cycle “planning-deployment-maintenance”. It should be noted that the considered model and algorithms describe one of the simplest backup options for reliable operation, which does not consider the possible effects of the physical environment on the network, as well as cyber-attacks, which will increase the probability of failures. These aspects can be considered by developing a more complex model for calculating the intensity of UAV failures.

## 7. Conclusions

The main contribution of this research is UAV deployment strategies and algorithms for forming the flying network, ensuring the dependable transmission of data from sensors or other sources of information located in dangerous or hard-to-reach places to the crisis centre, and models for ensuring continuous operation with given reliability.

The three UAV deployment strategies for forming the flying network are proposed as follows:

- Strategy for the route that involves the movement of each UAV to the first point of the LiFi route and the further deployment of the network within it;
- Radial movement strategy that consists of the movement per each UAV immediately to the destination point on the LiFi route;
- A strategy for the middle point of the route involves moving each UAV to a point as close as possible to the middle of the LiFi route and further deploying the network within it.

This article presents the developed software tool “Simulation Way” to support the planning of UAV deployment for forming the LiFi network, which has a three-level architecture and consists of the following parts: (i) GUI; (ii) business logic for interaction between data storage and GUI and contains report generation modules (results), calculation core modules, and external algorithm modules that can interact with each other; and (iii) storage data for receiving and storing in the form of data files the results of calculations and reports on the process of the software tool. In addition, many settings are available to the crisis centre operator using the “Simulation Way” software: set the dimensions of the working area of the production room, generate the required number of obstacles with given characteristics, and choose methods (rules) for bypassing these obstacles. Moreover, input data generated during one simulation iteration can be used in subsequent iterations.

The developed software tool “Reliability Level” results were also presented. Crisis centre operators can use this tool to determine the time of operation for the flying LiFi network with a reliability level not less than the minimum permissible for a given time. The software tool “Reliability Level” has a single-level structure and a monolithic module that combines all the logic.

The crisis centre operator uses the console interface to interact with the “Reliability Level” software tool. That operator can enter the following parameters: (i) UAV failure rates; (ii) setup time of the flying LiFi network; (iii) operation time of the flying LiFi network in data transmission mode; (iv) time resource of the on-board UAV battery; and (v) the minimum acceptable level of reliability with which a flying LiFi network can operate.

The proposed complex of strategies, algorithms, and software tools forms a decision-support system, which was created as part of the project to develop unmanned systems for ensuring monitoring and communications in conditions of destruction and external influences.

Further research can be aimed at expanding the strategies for deploying and supporting the reliability of flying networks and developing algorithms for their evaluation and selection depending on requirements and limitations, as well as their compatible application with intelligent robotic systems for cleaning dangerous spaces [68]. In addition, studies that consider using heterogeneous UAVs and robots for such systems may also have a sense [69].

Moreover, the software or software packages that would solve the discussed problems in the paper are unavailable on the market. This is due to certain specifics of these tasks and the novelty of the proposed methods. The capabilities of the developed software tools can be expanded by using modern tools available on the market:

- Matlab/Simulink and OptiSystem—for modelling optical communication systems, including LiFi;
- Ekahau and iBwave—for planning and optimising LiFi networks;
- AutoCAD/Revit and SketchUp—to create accurate 3D models of premises, plan placement of transmitters in them, and estimate coverage;
- LightTools and TracePro—for modelling optical systems and tracing light rays;
- NS3 (Network Simulator 3) and OMNeT++—for LiFi network optimisation and resource management;
- Arduino/Raspberry Pi and Osram Opto Semiconductors—for prototyping and testing LiFi hardware.

## Figures and Tables

**Table 1:** Data characterising the process of deploying the flying LiFi network according to the strategy of the first point of the route with different speeds.

UAV Number in the Queue	Distance to the Specified Point on the LiFi Route, m	UAV Placement Time on the LiFi Route, s			The Time of Setting Up the Flying LiFi Network s	Deployment Time of the Flying LiFi Network, s		
		Speed of 2 m/s	Speed of 3 m/s	Speed of 4 m/s		Speed of 2 m/s	Speed of 3 m/s	Speed of 4 m/s
UAV 1	25.99	13.00	8.66	6.50	30	43.00	38	36.5
UAV 2	23.99	12.00	8.00	6.00				
UAV 3	15.59	7.80	5.20	3.90				
UAV 4	10.34	5.17	3.45	2.59				
UAV 5	8.34	4.17	2.78	2.09				

**Table 2:** Data characterise deploying the flying LiFi network according to the route’s radial movement strategy at different speeds.

UAV Number in the Queue	Distance to the Specified Point on the LiFi Route, m	UAV Placement Time on the LiFi Route, s			The Time of Setting Up the Flying LiFi Network, s	Deployment Time of the Flying LiFi Network, s		
		Speed of 2 m/s	Speed of 3 m/s	Speed of 4 m/s		Speed of 2 m/s	Speed of 3 m/s	Speed of 4 m/s
UAV 1	22.76	11.38	7.59	5.69	30	41.38	37.59	35.69
UAV 2	19.86	9.93	6.62	4.97				
UAV 3	12.82	6.41	4.27	3.21				
UAV 4	8.89	4.45	2.96	2.22				
UAV 5	8.34	4.17	2.78	2.09				

**Table 3:** Data characterise deploying a flying LiFi network according to the route midpoint strategy at different speeds.

UAV Number in the Queue	Distance to the Specified Point on the LiFi Route, m	UAV Placement Time on the LiFi Route, s			The Time of Setting Up the Flying LiFi Network, s	Deployment Time of the Flying LiFi Network, s		
		Speed of 2 m/s	Speed of 3 m/s	Speed of 4 m/s		Speed of 2 m/s	Speed of 3 m/s	Speed of 4 m/s
UAV 1	23.22	11.61	7.74	5.81	30	41.61	37.74	35.81
UAV 2	21.22	10.61	7.07	5.31				
UAV 3	26.47	13.24	8.82	6.62				
UAV 4	21.22	10.61	7.07	5.31				
UAV 5	12.82	6.41	4.27	3.21				

**Table 4:** Advantages and disadvantages of each strategy.

Strategy	Advantages	Disadvantages
The first point of the route	1. This strategy is beneficial when there are significant obstacles on the routes on which the UAVs should travel from the depot to all points on the LiFi route except the first one. By moving to the first point of the LiFi route, the UAV can avoid complex or risky paths requiring detours, ensuring a more straightforward and safer deployment. 2. The UAVs follow a predetermined path from the first point to the destination, making route planning and navigation simpler, especially inside production premises with complex structures.	1. Since each UAV has to move from the first point to its destination, this could lead to longer deployment times, especially if the first point is far from the depot. 2. The UAVs might consume more energy if the first point is far from the depot or if the overall route is longer, reducing the UAVs’ operational time and possibly requiring more frequent recharging or replacements.
Radial movement	1. UAVs move directly to their destination points on the LiFi route, reducing travel time and enabling faster LiFi network deployment. 2. This strategy is highly efficient inside production premises with minimal obstacles, as UAVs can take the most direct path, optimising both time and energy consumption.	1. In environments with many obstacles, this strategy may require complex navigation or might not be feasible, leading to potential delays or failed deployments. 2. Direct paths might expose UAVs to more risks, such as collisions or interference, especially in cluttered environments.
Midpoint strategy	1. This strategy balances the deployment process by moving UAVs to the midpoint of the LiFi route, potentially reducing the overall deployment time since UAVs can move in both directions along the route. 2. The strategy reduces the likelihood of congestion or queues at the midpoint, leading to a more efficient deployment process, especially when multiple UAVs are involved.	1. The need to coordinate UAV movements from the midpoint in two directions can complicate the deployment process, requiring more sophisticated planning and control mechanisms. 2. UAVs might need to cover more area compared to the radial strategy, which could lead to higher energy consumption, especially if the midpoint is far from the depot.

## Data Availability

Data is contained within the article.

## Abbreviations and Acronyms

The following abbreviations and acronyms included in the text are reported alphabetically:

Abbreviation/Acronym	Meaning
CC	Crisis centre
FANET	Flying ad hoc networks
FFO	Probability of failure-free operation
GPS	Global Positioning System
GUI	Graphical user interface
LiFi	Light fidelity
RF	Radio frequency
UAV	Unmanned aerial vehicle
WiFi	Wireless fidelity
